# Significant hyperbilirubinemia among well neonates due for discharge at Kawempe-Mulago Hospital, prevalence, factors associated, and accuracy of transcutaneous bilirubinometry for screening

**DOI:** 10.4314/ahs.v22i2.61

**Published:** 2022-06

**Authors:** Carolyn Nassuna, Abdallah Yaser, Charles Karamagi, Jamir Mugalu

**Affiliations:** 1 Department of Paediatrics and Child Health, Makerere University College of Health Sciences, Kampala, Uganda; 2 Mulago National Referral hospital, Kampala, Uganda

**Keywords:** Significant Hyperbilirubinemia, transcutaneous bilirubinometer

## Abstract

**Background:**

Neonates in low resource settings with a lack of pre-discharge screening and early intervention are at risk for complications associated with significant hyperbilirubinemia (SHB).

**Objectives:**

To determine the prevalence, factors associated and performance of transcutaneous bilirubin (TCB) in identifying well neonates with SHB.

**Methods:**

Over a one month period 235 well neonates 24 to 72 hours of age due for discharge at Kamempe-Mulago Hospital were enrolled in this study. Visual inspection using Kramer rule, transcutaneous bilirubin over the sternum using Draeger JM103 bilirubinometer, and serum bilirubin were determined. Neonates with SHB (total serum bilirubin warranting treatment) were referred for treatment. Relevant data were analyzed. A P-value of <0.05 was considered significant at 95% confidence interval.

**Results:**

Thirty two (13.6%) of the neonates had SHB and three (1.3%) had levels above exchange transfusion threshold. Significant hyperbilirubinemia was independently associated with CRP ≥ 10mg/l (AOR 3.96, CI 1.23–12.73, p 0.021), ABO discordance (AOR 3.67, CI 1.28–10.49, p 0.015), jaundice in a previous sibling (AOR 3.565, CI 1.10–11.51, p 0.034) and time of first feed > 1 hour (AOR 2.74, CI 1.10–6.90, p 0.007). The sensitivity, specificity, positive and negative predictive values of TCB were 96.5%, 84.6%, 47.5% and 99.4% respectively compared to 31.2%, 98.5%, 76.9% and 90% respectively for visual assessment (Kramer grading).

**Conclusions:**

A significant number of well neonates have SHB. Transcutaneous bilirubinometry is a suitable screening tool in this setting. Early initiation of feeding should be promoted. The cause for high CRP among well neonates with SHB needs to be studied further.

## Background

Jaundice affects 60% to 80% of all neonates the majority of whom no intervention is needed, but approximately 5–15% will develop significant jaundice/hyperbilirubinemia (Serum bilirubin >95^th^ percentile on Bhutani hourly specific bilirubin nomogram) to warrant close monitoring and/or treatment^1^–^4^. Underlying conditions like prematurity, sepsis, blood group incompatibility, G6PD deficiency, inadequate feeding among others may cause a rapid rise of bilirubin leading to severe neonatal jaundice (SNJ) (Significant jaundice with acute neurologic changes or needing exchange blood transfusion) necessitating hospitalization^5^,^6^.

Severe neonatal jaundice is among the leading causes for hospitalization in the first month of life 6–8. Neonates with SNJ may progress to brain dysfunction (acute bilirubin encephalopathy (ABE)) and death while those who survive following ABE are at risk for disabilities^9^,^10^. In the developed countries systematic risk assessment for SNJ, bilirubin level estimation and timely intervention have considerably reduced SNJ, need for exchange transfusions and SNJ associated disabilities 11,12.

Globally SNJ affects 481000 term/ near term neonates annually; of these 114000 die and 63000 survive with disability 13. The majority of such neonates are in Low and Middle-income countries (LMICS) due to health system bottlenecks that hinder screening and early treatment 6,7,13,14. Whereas the WHO recommends 24-hour hospital stay after uncomplicated delivery, pre-discharge jaundice assessment, 72 hours and day 7 reviews^15^; implementation of these recommendations has been challenging in LMICS for various reasons.

At Kawempe-Mulago hospital (KMH) with its high patient volume; well neonates are discharged by 6–12 hours of age and are not screened for jaundice. In view of the consequences associated with SHB; we conducted this study at KMH to determine the prevalence of SHB and associated factors among well neonates so as to define the magnitude of the problem and advice policy. This high patient volume and short hospital stay renders serum bilirubin screening not feasible so we assessed the performance of transcutaneous bilirubinometry and visual inspection as alternatives screening methods.

## Methods

### Study Setting

This study was conducted at the postnatal ward of the Kawempe-Mulago National Referral Hospital (KMH). Kawempe-Mulago Hospital is Uganda's National Referral Hospital and the training hospital for Makerere College of Health Sciences it records 31000 deliveries annually.

After delivery well neonates are kept by the mother's side in the postnatal ward where the mother-baby pair is observed for 6–12 hours following normal vaginal delivery and 72 hours following cesarean delivery.

In the postnatal ward exclusive breastfeeding is encouraged and health education given to the mothers. The neonates are assessed by midwives and initial vaccines (BCG and Oral Polio) are administered. Unwell neonates are reviewed by a doctor and transferred to the Special Care Baby Unit (SCBU) for further care if needed. Well neonates continue rooming-in with their mother until discharge. At discharge parents are advised to take the baby to a nearby health facility after one week for review. The postnatal ward does not have a structured protocol for review of the neonate.

### Study Design

This was a cross-sectional study.

### Study Population

Well neonates 24 to 72 hours of age due for discharge and whose parents have consented to participate in the study. A well neonate was one reported by the midwife to have no concerns and is awaiting discharge.

### Sample size

Because we recruited neonates ≥ 24 hours of age, the majority of our study participants were born by cesarean section since most vaginally delivered babies would have been discharged by then. With approximately 31000 annual deliveries and 25% cesarean section rate^16^, the available population to us over 1 month period was approximately 646. Estimated sample size from the available population considering a prevalence for significant hyperbilirubinemia in Kenya of 34%(8) using Open-Epi for population survey at 95% CI, was 227 babies. Assuming a 3.5% for the incompleteness of data our final sample size was 235.

### Study Procedure

Parents or primary caregivers of the neonates who meet study criteria were given information about the study and informed consent was obtained by the principal investigator/research assistants. History was taken and study neonates examined by the principal investigator or research assistant.

Gestational age was determined using mother's records where available and the New Ballard Score^17^ conducted by the trained research assistant. Anthropometries were taken. Data capturing sheet was used to enter the relevant information.

Risk factors for SHB considered in the datasheet included blood group, mode of delivery, gestational age, the risk for sepsis (rapture of membrane >18hours, maternal intrapartum fever, and offensive liquor), history of jaundice in a previous child, time of initiating feeds, bruising/cephalohematoma and weight loss (≥ 75th percentile in the application tool for monitoring weight loss in newborn^18^).

Study neonates were assessed for jaundice by visual inspection and graded as per the Kramer rule^19^, we set Kramer grade 0,1,2,3,4 and 5 to correspond to bilirubin levels <80µmol/l, 80–100µmol/l, 101–150umol/l, 151–200µmol/l, 201–250µmol/l and > 250µmol/l respectively. Visual assessment was done by a trained research assistant just prior to blood sampling. After the research assistant determined the Kramer score the principal investigator repeated the score. If there was disagreement between the two scores, the Kramer-core obtained by the principal investigator was taken as final. Transcutaneous Bilirubin (TCB) measurement was done by the principal investigator using a calibrated Draeger JM103 transcutaneous bilirubinometer over the sternum. We ensured the interval between transcutaneous bilirubinometry and blood sampling was not >30 minutes.

For all study neonates 1mls of blood was collected by venipuncture for total serum bilirubin, blood grouping and C-reactive protein. The blood collection sites were cleaned with 70% alcohol and allowed to air dry. Blood samples were dispensed into EDTA-free containers, placed in biohazard bags then placed in a transport box to minimize direct light exposure. The samples were transported within one hour to Mulago chemistry laboratory. Total serum bilirubin was determined by the diazo colorimetric method using COBAS 6000 bilirubin analyzed. The laboratory calibrates the COBAS 6000 machine on a daily basis to ensure the validity of results.

Mother's documented blood group was ascertained and for those with no blood group documented; blood grouping was done.

Neonate found to have SHB i.e any level 20µmol/l within treatment threshold or greater on the age specific bilirubin nomogram (Using the neonatal academic hospitals' consensus guidelines for South African hospitals and primary care facilities)^20^, elevated CRP >10mg/dl and those with fever were referred to the SCBU for further management.

### Data management and analysis

Information captured in the datasheet was checked by the principal investigator for completeness and accuracy. Data were entered into the computer using Epi-data version 3.0 and thereafter exported into STATA version 14.0 for analysis with the help of a statistician. The baseline characteristics of study participants were summarized in a table. The prevalence of SHB was computed as a ratio of neonates with total serum bilirubin warranting treatment to the total number of study neonates. Univariate analysis was conducted for continuous variables. Multivariate analysis for factors associated with SHB was determined in a stepwise manner.

A P-value of < 0.05 was considered significant and confidence interval of 95% was used. Through a 2x2 table; the performance of transcutaneous bilirubinometer and visual assessment were determine. The sensitivity, specificity, positive and negative predictive values were computed. Results were summarized in tables.

## Results

Between 29st March 2017 and 3^rd^ May 2017 a total of 235 neonates were enrolled. The average age at enrollment was 49 (±14.4) hours. The median birth weight was 3.15kg (range 1.9–4.6kg). Sixty seven neonates delivered vaginally were enrolled; these neonates were not yet discharged because of maternal health concerns.

The majority were exclusively breast fed (96%). Of the vaginal delivered 55/67 (82.1%) breast fed within the first hour compared to 83/168 (49.4%) by cesarean section. Thirty eight neonates had excessive weight loss, of these 19/138 fed < 1 hour and 19/97 fed ≥ 1 hour after birth. Thirty two (13.6%) of the neonates had SHB (Table 1); 15 had TSB above phototherapy threshold, 14 had ≤ 20µmol/l below phototherapy threshold and 3 above exchange transfusion threshold. The mean TSB among neonates with SHB was 240µg/l (range132–481µmol/l). Twenty two neonates had elevated CRP; 9 had fever but 13 were well with no risk for sepsis. Visible jaundice was apparent in 43(18.3%) neonates graded as Kramer grade 3, 2 and 1 in 6, 7 and 30 neonates respectively.

**Table 1:** Baseline characteristics and factors associated with significant hyperbilirubinemia

VARIABLES	Significant hyperbilirubinemia		OR (95%CI)	p-value
	No (203)	Yes (32)		
**Sex**				
Male	108(53.2)	18(56.2)	1.13(0.53–2,39)	0.748
Female	95(46.8)	14(43.8)		
**Gestational age**				
≤37+6	28(13.8)	7(21.9)	1.85(0.73–4.67)	0.193
≥38	175(86.2)	25(78.1)		
**Birth weight**				
<2.5kg	14(7.0)	3(9.4)	1.39(0.37–5.15)	0.616
2.5–3.9kg	179(88.1)	25(78.1)		
≥4kg	10(4.9)	4(12.5)	2.75(0.80–9.39)	0.105
**Jaundice in previous** **baby**				
Yes	15(7.4)	7(21.9)	3.50(1.30–9.44)	**0.013**
No	188(92.6)	25(78.1)		
**Time of first feed**				
≤1 hour	124(61.1)	14(43.8)		
>1 hours	79(38.9)	18(56.2)	2.25(1.05–4.79)	**0.036**
**Excessive weight loss** *				
Yes	35(19.1)	3(11.1)	0.5(0.14–1.79)	0.294
No	148(80.9)	24(88.9)		
**Rupture of membrane** **(ROM)**				
≥18hrs	11(5.4)	1(3.1)	0.56(0.07–4.51)	0.589
<18hrs	192(94.6)	31(96.9)		
**Mother-infant ABO** **discordance**^a^				
Yes	21(12.4)	9(31.0)	3.17(1.27–7.87)	**0.013**
No	148(87.6)	20(69.0)		
**Mother-infant Rh** **discordance**^a^				
Yes	4(2.4)	2(6.9)	3.05(0.53–17.5)	0.22
No	165(97.6)	27(93.1)		
**C-reactive protein** a				
<10	151(91.5)	22(73.3)		
≥10	14(8.5)	8(26.7)	3.92(1.47–10.42)	**<0.006**
**Mode of delivery**				
Caesarean section	141(69.5)	27(84.4)	2.14(0.78–5.85)	0.136
Vaginal delivery	62(30.5)	5(15.6)		
**HIV exposure**				
Yes	23 (11.3)	1(3.1)	0.18(0.03–1.93)	0.252
No	180(88.7)	31(96.9)		

* 25 neonates did not have birth weights

a Missing lab results were excluded

From the univariate analysis factors 4 factors with p<0.2 and scientific rational were included in the multivariate analysis to determine factors independently associated with SHB see Table 2.

**Table 2:** Factors independently associated with significant hyperbilirubinemia

Variables	Adjusted Odds Ratio	95% Conf. Interval	P-value
**Jaundice in previous baby**			
No	1		
Yes	3.565	(1.10–11.51)	**0.034**
**Time of First feed <1hr**			
No	2.74	(1.10–6.90)	**0.007**
Yes	1		
**C-reactive protein**			
<10	1		
≥10	3.962	(1.23–12.73)	**0.021**
**ABO discordance**			
No	1		
Yes	3.670	(1.28–10.49)	**0.015**

To elaborate on how well visual assessment identified the level of TSB we categories our neonates in to 6 groups based on TSB range; along with this we described how they had been categories by visual assessment see table 3. Only 110 Kramer scores obtained by principal investigator and 104 by research assistant correctly predicted TSB level. Visual assessment correctly identified majority of neonates with TSB <100µmol/l but with increasing TSB level visual assessment became less accurate.

**Table 3:** Total serum bilirubin and Kramer grading of study neonates

TSB level (µmol/l)	Number of neonates Per TSB level	Number of neonates and Kramer grading (corresponding serum bilirubin in µmol/l)					
		0 (<80)	1 (80–100)	2 101–150)	3 (151–200)	4 (201–250)	5 (>250)
**<80**	83	82			1		
**80–100**	27	0	27				
**101–150**	65	53	12				
**151–200**	39	26	9	3	1		
**201–250**	11	3	5	2	1		
**>250**	10	1	4	2	3		

To determine the performance of TCB and visual assessment as screening modalities with TSB as the gold standard we categories neonates who were correctly assigned treatment as compared to the TSB. TCB correctly identified the majority of neonates who needed phototherapy 28/32 (87.5%) while visual assessment correctly identified the majority of those who did not need phototherapy 200/203(98.5%).

## Discussion

The prevalence of significant hyperbilirubinemia within the first 72 hours of life in our study was 13.6% with three neonates (1.3%) having serum bilirubin levels above exchange transfusion threshold. This prevalence seems lower than that observed in the Kenyan study8 from which we derived our sample size although this could be because they included neonates up to 28 days of life.

A study conducted in Egypt looking at predictors of significant jaundice among well neonates without risk factors in the first 5 days found a prevalence of 16% (3), which was slightly higher than ours but we attribute this to their recruiting neonates beyond 72 hours of age.

From our literature search we noted very scanty literature on hyperbilirubinemia among well neonates in the first 72 hours of life from LMICs. When we compare our prevalence to those observed in other studies, from which SHB in the first 72 hours could be derived^21^–^23^, our prevalence was higher; it should be noted that of the 32 neonates with SHB in this study 14 had TSB below phototherapy threshold but by the South African guidelines which we used these neonates are categorized as having SHB.

Three (3) of the neonates in this study had bilirubin levels at exchange transfusion threshold with serum bilirubin levels of 332.3, 420 and 481.4µmol/l before 60 hours of life. These are examples of neonates in LMICs likely to be discharged home only to suffer consequences of SHB due to lack of risk assessment and predischarge screening.

Thirty blood group O mothers gave birth to neonates with a different blood 30/198 (15.1%) this finding is similar to that observed in Nigeria^24^. Nine of the neonates born to these 30 mothers developed SHB. ABO discordance was independently associated with SHB (AOR 3.6, CI 1.28-–0.49 p0.015) (Table 2). This finding suggests possibility of ABO associated hemolysis among causes of SHB in this study and this is in line with findings from a systematic review looking at risk factors for severe neonatal jaundice in LMIC5.

Rhesus incompatibility is a known risk factor for SHB^5^,^13^. In this study 6 mothers-infant pairs had Rh discordance from whom 2 neonates developed SHB. Of the 6 mothers 2 were multiparous and aware of their rhesus status and had made arrangements for anti D while the other 4 of whom 2 were multiparous and 2 primeparous were not and we advised them to get. Whereas the odds of developing SHB was high in the univariate analysis (OR3.05, CI 0.53 -17.5, p0.22) (Table 1), it was not statistically significant. This observation could be due to the small number of Rh discordant neonates. We did not run direct coombs test hence we cannot infer to rhesus incompatibility.

Elevated CRP ≥10mg/l was independently associated with SHB (AOR 3.96, CI 1.2–12.7 p 0.021) (Table 2). Of the 22 neonates with elevated CRP; 8 had SHB. None of the neonates with elevated CRP and SHB were unwell, 3 had ABO discordance while for the 5 no identifiable reason for SHB could be ascertained.

Whereas perinatal stressors including prolonged labour, meconium aspiration, birth asphyxia, intraventricular hemorrhage, induction of labour may cause an elevation in CRP in the first few days after birth^25^–^27^ in view of the high mortality associated with neonatal jaundice in the first 6 days of life^14^ and sepsis as one of the major contributors to neonatal mortality in LMIC^28^, we concluded that sepsis was most probable cause for these findings. This finding of seemingly well neonate due for discharge having elevated CRP and SHB is a phenomenon that needs further review.

Neonates whose mothers have had previously infants with jaundice were more likely to have SHB 7/22 (AOR 3.5, CI 1.10–11.5, p 0.034) (Table 2), of the 7 neonates with history of jaundice in previous sibling 5 had ABO/Rh discordance while the other two no reason could be identified. Other familial causes of neonatal jaundice including G6PD deficiency, hemoglobinopathies and enzymopathies could explain this occurrence^5^. History of jaundice in previous infant is a known risk factor for jaundice in subsequent infant and should always be asked^1^.

The neonates in this study whose first feed were ≥ 1hour after birth were more likely to develop SHB (AOR 2.74, CI 1.1–6.9 p 0.007) (Table 2). This finding is similar to that observed in the Zimbabwean study that looked at early initiation of breastfeeding on jaundice^29^. Inadequate enteral feeds favors increased entero-hepatic circulation and jaundice^30^. The importance of early initiation of breastfeeding cannot be over emphasized. Of the 235 study neonates enrolled 210 had both birth weight and weight at enrollment verified. Thirty eight 38/210 (18.1%) had excessive weight loss. Only 3/38 of the neonates with excessive weight loss had SHB (Table 1), this finding is contrary to findings from other studies that have demonstrated high weight loss being associated with SHB^5^,^6^. The reason for our finding might lie in the fact that the number of neonates with excessive weight loss was small and our screening was done before the peak time which tends to occur between 3 and 5 days of life.

The majority of the neonates with SHB 12 (37.5%) the cause could not be ascertained. Whereas identification of the cause for significant hyperbilirubinemia is important in deciding the course of management and family counseling for future pregnancies, the cause may not be established in as high as 50% of cases^22^,^31^,^32^. Uganda has a high prevalence of G6PD deficiency^33^ and other hemoglobinopathies, it would be important to ascertain how much these contribute to SHB.

Transcutaneous bilirubin identified the majority of neonates with SHB 28/29 compared to visual inspection 10/32 (Table 4). This is in line with findings from other studies that found TCB to yield results that highly correlate with serum levels even among black African infants^35^,^36^. Transcutaneous bilirubinometry is steadily being accepted as modality for screening term neonates predischarge^1^,^34^ and can be easily adopted in our setting.

**Table 4:** Sensitivity, specificity and accuracy of TCB and Kramer grading

	Needed Phototherapy	TCB* Correctly assigned	TCB* Wrongly assigned	Kramer grading Correctly assigned	Kramer grading Wrongly assigned
**Yes**	32	28	31	10	3
**No**	203	171	1	200	22
**Performance of screening methods**					
	**TSB**	**TCB**		**Kramer grading**	
**Sensitivity** **%**	100	96.5		31.2	
**Specificity%**	100	84.6		98.5	
**PPV%**	100	47.5		76.9	
**NPV%**	100	99.4		90.0	

* TCB was obtained on 235 study neonates but only 231 used in the analysis because 4 TCB's were performed >30minutes after blood samples were drawn.

Visual assessment for jaundice is a very well described process but its accuracy is very subjective that even specialists using it wrongly predict bilirubin levels 6,34,37. From our study (Table 3) the majority of neonates with jaundice could be identified by visual inspection but grading of the jaundice was poor. Both the trained research assistant and the principal investigator through visual inspection tended to underestimate serum bilirubin level with 6 of the Kramer scores by the research assistant being lower than that obtained by the principal investigator. We observed with increasing serum bilirubin visual inspection poorly pedicted serum bilirubin level. The Kramer grading was found to have a very low sensitivity 31.2% (Table 4) we therefore do not recommend it for screening.

## Conclusion

Significant hyperbilirubinemia is prevalent among well neonates due for discharge. Transcutaneous bilirubinometry identifies majority of neonates with SHB. Delayed initiation of feeding is a risk factor for SHB. Observed phenomenon of well neonates with SHB and elevated CRP without identifiable reason needs to be explored.

## Study Limitation

The majority of the neonates were delivered by caesarian section hence our findings might not be very representative of neonates delivered vaginally. We could not do coombs test, blood cultures, screening for congenital infections like toxoplasmosis, rubella, cytomegalovirus, herpes, urinalysis, film comment and G6PD assay to ascertain other causes of significant hyperbilirubinemia. Total serum bilirubin tends to peak at 3 to 5days of age so some of our study infants might have developed significant jaundice after the study period.

What is already known? Screening neonates for jaundice is necessary in identifying those at risk for significant hyperbilirubinemia. Transcutaneous bilirubinometry is a good screening tool for hyperbilirubinemia.

What this study adds? In the first 72 hours of life 1 in every 10 neonates have significant hyperbilirubinemia and 1 in every 100 may have bilirubin level approaching exchange transfusion thresh hold. Delayed initiation of feeds increases risk for hyperbilirubinemia.

## List of abbreviations

Rh: Rhesus
ABO: Blood groups A, B and O
CRP: C-reactive protein
LMICs: Low and Middle Income Countries
SCBU: Special Care Baby Unit
SHR: Significant Hyperbilirubinemia
TCB: Transcutaneous Bilirubin
TSB: Total Serum Bilirubin
WHO: World Health Organization

## Declaration

### Availability of data and materials

The datasets used and/or analyzed during the current study are available from the corresponding author on reasonable request.
